# Ocular Surface Evaluation After the Substitution of Benzalkonium Chloride Preserved Prostaglandin Eye Drops by a Preservative-free Prostaglandin Analogue

**Published:** 2019

**Authors:** Nara Lidia Vieira LOPES, Carolina P. B. GRACITELLI, Maria Regina CHALITA, Nubia Vanessa Lima de FARIA

**Affiliations:** 1 Department of Ophthalmology and Vision Science, Glaucoma Service, Federal University of São Paulo, São Paulo, Brazil; 2 Department of Ophthalmology, Federal University of Brasilia, Brasilia, Brazil

**Keywords:** Glaucoma, Ocular Surface, Preservative-free Prostaglandin, Benzalkonium chloride

## Abstract

To evaluate ocular surface changes after withdrawal of Benzalkonium chloride (BAK) in patients with glaucoma in monotherapy with BAK-preserved prostaglandin. This was a prospective observational study. All patients underwent complete ophthalmologic examination and evaluation of ocular surface. A questionnaire was filled regarding symptoms of dry eye (Ocular Surface Disease Index [OSDI]) at the beginning of study. The treatment was switched to preservative-free tafluprost for 6 weeks and after this period, all patients were re-evaluated. All patients reported improvement of symptoms. The green lissamine test showed a significant improvement of the ocular surface, with most patients classified as light dry eye (P < 0.001). A significant improvement in the score (P < 0.001) was also found, with an average of 17.95 ± 5.35 points, which classifies the patients' symptoms in the normal to light zone. Benzalkonium chloride withdrawal reduced the signs and symptoms of dry eye in patients with primary open angle glaucoma (POAG).

## INTRODUCTION

Glaucoma is a chronic disease and the second leading cause of irreversible blindness in the world [1]. The main risk factor is intraocular pressure (IOP) and currently, the only treatable one. One of the first treatment options is drug therapy to reduce IOP, since the control of this factor delays the progression of glaucoma and consequently blindness. In the last decade, availability of prostaglandin analogous eye drops has facilitated the management of patients with glaucoma. They are currently the first drug of choice in the treatment of glaucoma due to potent IOP reduction, few adverse reactions, only daily application and good adherence. Although prostaglandin analogs are safe eye drops, they are known to have side effects related both to the specific properties of each analog, as well as to the preservatives used in these medications [2]. The well-known characteristic side effects of prostaglandins (PG) include iris pigmentation, eyelid pigmentation, eyelash extension and deepening of the upper eyelid sulcus [3]. Side effects commonly occur with PG-related drugs and other ophthalmic antiglaucomatous agents include ocular surface diseases (OSDs) such as tear reduction and superficial punctate keratopathy (SPK). In addition, to the ophthalmic antiglaucomatous agent itself, the effects of preservatives have been indicated as a causative factor of OSD associated with ophthalmic antiglaucomatous agent administration. [4] Preservatives are used to inhibit the growth of microorganisms in eye drops bottles, making safe the use of multi-drop vials. They also protect biodegradation and help maintain drug potency. Benzalkonium chloride (BAK) one of the most commonly used preservatives in antiglaucomatous medications, is a cationic surfactant that binds to the cell membranes of microorganisms, increasing permeability and consequently cell lysis. Previous studies have shown that BAK has a toxic effect on the ocular surface, as it has a detergent effect on the lacrimal layer of the tear film. Also, it has pro-inflammatory properties and induces apoptosis of goblet cells [5-7].

There are many studies indicating that more than 60% of patients with glaucoma have signs and symptoms of ocular surface disease (OSD). Therefore, the purpose of this study was to evaluate ocular surface changes after withdrawal of BAK in patients with glaucoma who use eyedrops of prostaglandin analogs preserved with BAK [8].

## METHOD

This prospective observational study adhered to the tenants of the Declaration of Helsinki and was approved by the Institutional Review Board. Additionally, a written informed consent was obtained from all participants. We enrolled patients with primary open angle glaucoma. The definition of glaucoma was based on the presence of repeatable (≥ 2 consecutive) abnormal standard automatic perimetry (SAP) test results on the 24-2 program of the VF (Humphrey Field Analyzer; Carl Zeiss Meditec, Inc.) or if progressive glaucomatous optic disc changes were noted on stereo photographs, regardless of the results of SAP. We have described abnormal SAP results as those with a pattern standard deviation index outside the 95% confidence limits and/or glaucoma hemifield test results outside the reference range. The inclusion criteria were age > 18 years, treatment for glaucoma with monotherapy prostaglandin for at least 6 months and symptoms of dry eyes. The exclusion criteria were Sjogren’s syndrome, Steven’s Johnson syndrome, ocular surgery in the last year including refractive surgery, allergy to some component of eye drops, pregnancy, systemic diseases affecting the ocular surface or any systemic medication affecting the ocular surface.

All patients underwent complete ophthalmologic examination at the beginning of the study (visual acuity without and with correction, biomicroscopy, eyelid examination, conjunctiva, cornea and tear film), complete anamnesis where the complaints were recorded. However, a questionnaire of ocular surface disease was filled for each patient to evaluate the impact of ocular surface changes on daily activities. The diagnostic tests used in this study were Schirmer's test, Green lissamine test, tear film break-up time (BUT) and Goldmann applanation tonometry. Tear film BUT was measured following the guidelines described in the Dry Eye Workshop (DEWS) report [9]. Staining of the cornea with lissamine green was performed and graded according to the Bijsterveld’s scale [10]. Schirmer I test without anesthesia was performed following the guidelines published in the DEWS report [11]. Schirmer paper strips were inserted in the eye over the lower lid margin, midway between the middle and the outer third.

The patient was asked to close the eye, and after 5 minutes, the wetting of the Schirmer paper was measured. Intraocular pressure measured with a slit lamp–mounted Goldmann applanation tonometer. Dry eye symptoms were assessed using the Ocular Surface Disease Index (OSDI). The questionnaire underlying the OSDI is specifically designed for patients with dry eye syndrome and asks patients regarding the frequency of specific symptoms and their impact on vision-related functioning. After the exams, patients received sufficient free samples of tafluprosta (Saflutan) eye drops for the use of a drop once overnight for 6 weeks. Such samples were provided by the examiner to avoid discontinuation of treatment for socioeconomic reasons.

**Figure 1:**
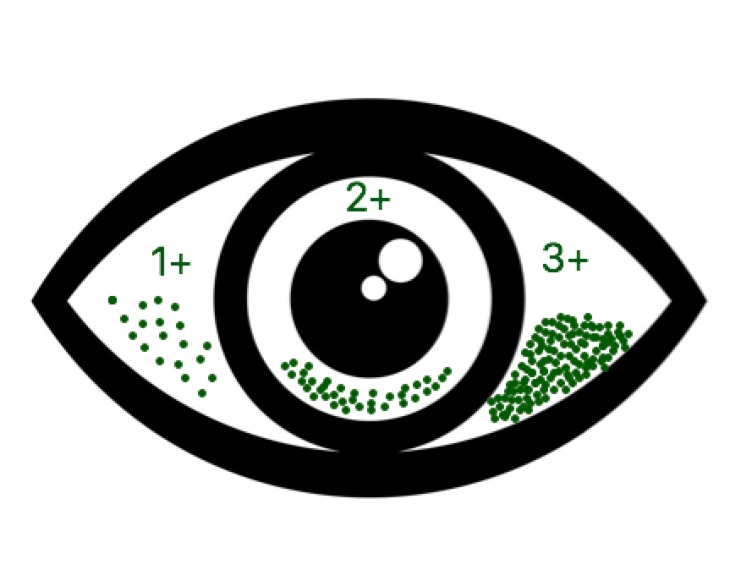
Van Bijsterveld‘s Scale

After 6 weeks, all patients underwent a new evaluation with the same exams and questionnaire. In this work, the diagnosis of dry eye was established as tear film BUT less than 5 seconds, Schirmer test less than 5 mm and / or Green lissamine test greater than 3 according to the van Bijsterveld scale (0 to 9) [9] (Fig 1).

Descriptive statistics were calculated for demographic and clinical characteristics. The means and standard deviations (SD) presented for the normally distributed variables and medians and interquartile ranges presented for non-normally distributed variables. Paired t-test was used to compare differences in the scores before and after the medication change. All statistical analyses were performed with Stata computer software (version 13; StataCorp LP, College Station, Texas, USA). The alpha level was set at 0.05.

## RESULT 

Eleven patients (22 eyes) were evaluated, 8 women and 3 men. All patients had the diagnosis of primary open-angle glaucoma and used BAK preserved prostaglandin analog eye drops as monotherapy for at least one year. The mean age was 64.9 ± 6.07 years. In this sample, 6 patients used bimatoprost 0.3%, 2 Travoprost 0.004% and 3 latanoprost 0.005%. At the beginning of the study, all patients had a dry eye diagnosis with at least 2 diagnostic exams with altered results and had an important complaint of foreign body sensation and burning eyes. The Schirmer test had a mean score of 5.0.9 ± 2.75 mm (range 1-10 mm); The tear film BUT presented an average of 6.68 ± 2.07 seconds (range 3-10 seconds); The green lissamine test was a moderate to severe dry eye in 15 eyes with a Bijsterveld index with a mean of 6.27 ± 2.72 (range 1-6). Intraocular pressure showed an average of 11.5 ± 1.56 mmHg (range 9 - 14mmHg). All patients answered 10 questions of the OSDI questionnaire, since 2 questions did not apply to any patient in the study. The average score obtained in the analysis of the questionnaire was 35.27 ± 10.67 points (range 17.5 - 60 points), characterizing the patients' symptoms in mild to moderate.

After 6 weeks, all patients were re-examined. All reported improvement of symptoms and only 4 patients complained of a slight sensation of sand in the eyes. The Schirmer test presented a mean ± SD of 4.36 ± 2.40 mm (range 1-12 mm) without significant improvement (P = 0.198); tear film BUT score had a mean ± SD of 5.5 ± 2.15 seconds (range 3-10 seconds) without significant improvement (P = 0.113); the green Lissamine test showed a significant improvement of the ocular surface, with most patients classified as light dry eye (P < 0.001). The Bijsterveld index presented a mean ± SD of 3.04 ± 1.25 (range 1-5). A significant improvement in the score (P < 0.001) was also found, with a mean ± SD of 17.95 ± 5.35 points (range 5-25 points), which classifies the patients' symptoms in the normal to light zone.

## DISCUSSION

This study demonstrated a significant improvement in OSDI and in van Bijsterveld’s scale in patients who were using PG with BAK replaced by PG without BAK. This is the first study to evaluate ocular surface using OSDI and clinical evaluation in patients using PG with and without BAK. 

Currently, the only accepted way for preserving visual function in patients with glaucoma is reducing IOP. Although the disease is multifactorial, IOP remains the only modifiable risk factor for prevention or delay of visual deterioration. Chronic use of hypotensive eyedrops is associated with several adverse effects such as allergies, conjunctivitis, contact dermatitis, punctate keratitis and even failure of filtering surgery. This toxicity appears to be more associated with the preservative BAK than with the active component of the medication. From this point of view, single-dose preservative-free preparations would be a viable alternative to the currently used multiple-dose drops of eyedrops [12, 13]. This study demonstrated a significant improvement in OSDI questionnaire score and in lissamine green score in patients who were using PG with BAK replaced by PG without BAK. The decrease in ocular surface staining and reduction in the symptoms as observed in our study is in accordance with previous studies, which demonstrated that BAK is one of the major triggers that induces OSD in patients with long-term glaucoma therapy [1, 8, 9, 14] and the adverse effects are reversible, as we found replacing preserved to preservative free topical anti-glaucoma therapy.

Furthermore, we found a considerable improvement in self-reported quality of life of patients with glaucoma in accordance with previous studies that analyze symptoms and signs with preserved and preservative-free glaucoma medications in general [7, 15, 16]. Although, most studies use a washout period in this study, patients had straight substituted treatment from preserved to unpreserved prostaglandins, resembling daily clinical practice. Replacement of eyedrops without washout had no impact on IOP-lowering efficacy in patients, which were well controlled before study in accordance with previous studies [3, 7, 14].

In patients with glaucoma, tear dysfunction is mainly attributed to chronic administration of preserved glaucoma medications. Benzalkonium chloride is known to damage the ocular surface, reduce the density of epithelial and goblet cells and alter the lipid layer. These changes result in an impaired tear film with excessive evaporation [17]. There are different findings in symptoms and signs of dry eye between glaucoma-treated patients and controls in many studies. Van Went et al. [18] found that only tear film BUT and fluorescein staining grade were significantly altered in patients with treated glaucoma compared to untreated control group, and there was no significant difference for Schirmer test and OSDI. Some studies [16, 19] also reported significantly reduced tear film BUT and Schirmer test. Uusitalo et al. [20] showed a significant increase in tear film BUT after the substitution from preserved latanoprost to preservative-free tafluprost suggesting that tear film stability is increased after withdrawal of preservative, but not observed in this study. This finding might be explained by the factors that influence the reproducibility of these tests, like natural fluctuations during the day, different populations, variations in measurement techniques and scoring, systemic medications, environmental differences [21] or it could be also consequence of the small sample size and the absence of other tests to evaluate the tear film like osmolality and tear film thickness.

A significant improvement was seen in the OSDI as a reduction in complaints about symptoms related to ocular surface. This result associated with a decrease in ocular surface staining, and consequently improvement of keratitis, as observed in our study may suggest that avoiding BAK exposure improves the health of the ocular surface. 

This study had some limitations; the sample size was limited to 22 eyes. However, this is one of the first studies comparing two medications with and without BAK, therefore, these preliminary results confirm the effect of BAK in ocular surface of patients with glaucoma. Second, there was no randomization without mask and data collection was performed by only one examiner, which allows patient or observer bias, mainly at the assessment of OSDI symptoms. Besides, this was a cross-sectional study. Longitudinal studies have to be performed to evaluate the BAK effect during a certain period. Lastly, the effect of ocular surface in quality of life of patients with glaucoma was not assessed. Further studies would be necessary to measure the real impact of BAK effect in these patients. As glaucoma requires a continuous treatment, it is important to assure the adherence to the treatment. Nevertheless, some signs and symptoms of ocular surface disease, like conjunctival hyperemia, foreign body sensation or irritation difficult this adherence [22]. Once it could be avoided or get better, probably the overall glaucoma management and outcomes will improve.

## CONCLUSION

We found similar results to those found in the literature, which makes it possible to suggest that change of prostaglandin analogs BAK-preserved to tafluprost preservative-free is possible and can bring some benefits for patients like improvement of self-reported symptoms and reduction of clinical signs of dry eye in patients with glaucoma maintaining effective IOP control.
